# Long-term out of pocket expenditure of people with cancer: comparing health service cost and use for indigenous and non-indigenous people with cancer in Australia

**DOI:** 10.1186/s12939-019-0931-4

**Published:** 2019-02-12

**Authors:** Emily Callander, Nicole Bates, Daniel Lindsay, Sarah Larkins, Stephanie M. Topp, Joan Cunningham, Sabe Sabesan, Gail Garvey

**Affiliations:** 1 School of Medicine, Griffith University, Gold Coast, Australia; 2 Australian Institute of Tropical Health and Medicine (AITHM), James Cook University, Townsville, Australia; 3 College of Public Health, Medical and Veterinary Sciences (CPHMVS), James Cook University, Townsville, Australia; 4 College of Medicine and Dentistry (CMD), James Cook University, Townsville, Australia; 5 Menzies School of Health Research, Charles Darwin University, Darwin, Australia

**Keywords:** Cost, Rurality, Socioeconomic, Indigenous

## Abstract

**Background:**

Indigenous Australians diagnosed with cancer have poorer survival compared to non-Indigenous Australians. We aim to: 1) identify differences by Indigenous status in out-of-pocket expenditure for the first three-years post-diagnosis; 2) identify differences in the quantity and cost of healthcare services accessed; and 3) estimate the number of additional services required if access was equal between Indigenous and non-Indigenous people with cancer.

**Methods:**

We used CancerCostMod, a model using linked administrative data. The base population was all persons diagnosed with cancer in Queensland, Australia (01JUL2011 to 30JUN2012) (*n* = 25,553). Each individual record was then linked to their Admitted Patient Data Collection, Emergency Data Information System, Medicare Benefits Schedule (MBS), and Pharmaceutical Benefits Scheme (PBS) records (01JUL2011 to 30JUN2015). We then weighted the population to be representative of the Australian population (approximately 123,900 Australians, 1.7% Indigenous Australians). The patient co-payment charged for each MBS service and PBS prescription was summed for each month from date of diagnosis to 36-months post-diagnosis. We then limited our model to MBS items to identify the quantity and type of healthcare services accessed during the first three-years.

**Results:**

On average Indigenous people with cancer had less than half the out-of-pocket expenditure for each 12-month period (0–12 months: mean $401 Indigenous vs $1074 non-Indigenous; 13–24 months: mean $200 vs $484; and 25–36 months: mean $181 vs $441). A stepwise generalised linear model of out-of-pocket expenditure found that Indigenous status was a significant predictor of out of pocket expenditure. We found that Indigenous people with cancer on average accessed 236 services per person, however, this would increase to 309 services per person if Indigenous people had the same rate of service use as non-Indigenous people.

**Conclusions:**

Indigenous people with cancer had lower out-of-pocket expenditure, but also accessed fewer Medicare services compared to their non-Indigenous counterparts. Indigenous people with cancer were less likely to access specialist attendances, pathology tests, and diagnostic imaging through MBS, and more likely to access primary health care, such as services provided by general practitioners.

## Background

Aboriginal and Torres Strait Islander people (hereafter respectfully referred to as Indigenous Australians) have poorer survival after a cancer diagnosis compared to non-Indigenous Australians [1–4]. Between 2007 and 2014, five-year survival for Indigenous Australians was 50% compared to 65% for non-Indigenous Australians [1]. Indigenous Australians living in remote areas have much lower survival compared to those living in metropolitan areas [3, 5]. A number of studies have identified factors contributing to this survival inequality, including Indigenous Australians having increased risk factors for developing cancer [2], lower participation rates in national screening programs [2] and being more likely to be diagnosed with advanced disease [2, 4, 6].

Evidence also shows that there are differences in access to and uptake of treatment between Indigenous and non-Indigenous Australians [4, 7–9]. The reasons for this difference in access to and uptake of treatment are potentially complex and multifactorial, but include difficulty in geographical accessibility of services, and issues with the cultural and personal acceptability of services, in addition to clinical decisions about which options are offered to individuals [8, 9]. In order to develop the best treatment pathway for an individual, many clinical factors will be considered by the treating specialist. Indigenous Australians are more likely to be diagnosed with an increased stage of disease [4, 9], and have an increased number of comorbidities [4, 7, 9], which may alter the treatment options that are available or offered. These are “supply side” issues influencing the services that are accessed.

In addition to these supply side or clinical driven factors, there may be patient driven factors influencing treatment decisions. These are “demand side” issues influencing the services that are accessed. Physical location is an important consideration in the decision to access care for people living outside of urban areas. In rural or regional areas of Australia there are fewer oncology services available locally [10], and as such patients may be required to travel or relocate for treatment [11, 12]. Travel and accommodation for cancer patients comes at a high out of pocket cost [11], and there are large non-financial and other opportunity costs such as separation and isolation.

The overall cost of access to health care, through travel, accommodation, opportunity costs and the patient co-payment amounts often required to access healthcare outside of public hospitals in Australia, will influence demand for services. Previous research has indicated that, internationally, between 28 and 43% of cancer patients report financial distress or hardship as a result of expenditure on their cancer treatment (called “financial toxicity”) [13]. Such demand side access considerations may also be particularly important for Indigenous Australians, as a higher proportion of Indigenous Australians live in remote areas [14] and are more likely to be socioeconomically disadvantaged [15] and thus more likely to face higher transport costs for accessing care alongside a lower ability to pay. Understanding how much is paid for healthcare by people with cancer is an important part in understanding differences in access to care, as high out of pocket cost will act to reduce consumption of services.

Australia has a universal health care system and its public health insurance program, Medicare, provides free treatment at public hospitals, and free or subsidized medical treatment for care outside public hospitals [16]. Outside of public hospitals, if there is a difference between the amount the provider of a service charges and the rebate amount paid under Medicare, the patient is charged a co-payment. The Medicare system is designed to ensure that all people have access to the care they need, and that there is equity in payment for services so that the cost to the patient does not act as a barrier to accessing care [17]. However, for out-of-hospital medical services, the price set by service provider is unregulated, thus the charge to the patient is dependent on what the provider charges. The Pharmaceutical Benefits Scheme (PBS) is a part of Australia’s universal health system and seeks to provide affordable access to pharmaceuticals for Australians. The PBS contains a list of approved medications, which may be available at a subsidised rate (patients paying up to a set co-payment charge and the balance subsidised by the Australian government) with a valid prescription [16].

Australia has several policies which further assist individuals and family groups access healthcare by reducing the cost of individual services or the amount of overall expenditure. Once an individual or family group reaches a given level of out of pocket expenditure on co-payments during the calendar year, the individual or family group will have a higher proportion of their fees subsidized for the remainder of the year under the “Medicare Safety Net” scheme [16]. Furthermore, in 2010, the Closing the Gap (CTG) PBS Co-payment Programme was initiated to reduce the co-payment amounts for prescription medications to eligible Indigenous people living with, or at risk of chronic disease [18].^1^

Despite these policies to reduce costs to the patient for healthcare and the associated decline in demand for healthcare, 21% of people with cancer in Australia state that they skipped care due to the cost [19]. Within Australia, previous studies have quantified the out of pocket expenditure for breast cancer, prostate cancer and lymphedema patients, and patients living in rural areas [11, 20–24]. However, to date, none have sought to quantify the expenditure on healthcare for Indigenous people with cancer and none have sought to take into account the differences in access to health services when quantifying expenditure. Understanding the amount paid for healthcare by Indigenous people with cancer is important to determine the effectiveness of the current policies to minimise healthcare co-payments for Indigenous Australians, and also in ensuring that demand for healthcare is not reduced by high out of pocket cost.

The aims of this study were to: 1) identify if there is a difference in the long-term out of pocket healthcare expenditure on healthcare services incurred by Indigenous and non-Indigenous people with cancer; 2) identify whether there is any difference in the quantity and cost of individual services being accessed that may be contributing to the differences in total expenditure; and 3) estimate the number of additional health services that would be required to be supplied if access for different types of services was equal between Indigenous and non-Indigenous people with cancer.

## Methods

### Study design and participants

This study used a model of cancer costs based upon a whole of population linked dataset, CancerCostMod. The data linkage [25] and the development of CancerCostMod have been described in detail elsewhere [26]. Briefly, the base population of this dataset was a census of all patients diagnosed with cancer in Queensland, Australia, between 1 July 2011 and 30 June 2012, as recorded by the Queensland Cancer Registry (QCR) (*N* = 25,553). All cancer diagnoses in Australia, except for non-melanoma skin cancer are required by law to be recorded by the jurisdiction’s cancer registry. Each individual’s record was then linked to their Queensland Health Admitted Patient Data Collection (QHAPDC), Emergency Department Information System (EDIS), Medicare Benefits Schedule (MBS), and Pharmaceutical Benefits Scheme (PBS) records from 1 July 2011 to 30 June 2015.

### Socioeconomic status and rurality

We mapped the patient’s residential postcode at diagnosis to the Index of Relative Socio-Economic Disadvantage (IRSD) [27] and Australian Statistical Geography Standard (ASGS) [28], which were both developed by the Australian Bureau of Statistics’ (ABS). IRSD is a summary score of an area’s the economic and social conditions. Areas are assigned a score and then grouped into deciles ranked from highest to lowest economic and social status, as such it is a measure of relative disadvantage only. The authors then collapsed IRSD into quintiles, where Q1 was the most disadvantaged and Q5 was the least disadvantaged. The ASGS categorises remoteness into: major cities, inner regional, outer regional, remote, and very remote. The authors collapsed rurality into three categories: ‘metropolitan’, ‘regional’, and ‘remote’. Postcode was unknown for 151 records, and as such these were unable to be mapped to IRSD or ASGS.

### Indigenous identification

The original QCR dataset recorded whether people with cancer identified as Indigenous Australian or not for 87% of records. We used multiple imputation to impute the 13% of records with missing data for this variable, as described previously [26].

### Cost for total patient co-payment

The MBS and PBS datasets contained information on the total amount charged for the service or prescription, the Medicare rebate, and the patient co-payment. The patient co-payment was summed for MBS and PBS from the date of diagnosis for each month up to 36 months to give the expenditure for MBS and PBS separately. The patient co-payment was assigned a value of ‘0’ if the patient was alive, but had no recorded expenditure, and a missing value if the patient had died.

Descriptive analyses were performed to identify the relevant social and demographic characteristics of the sample (both weighted and non-weighted where appropriate), as well as the average annual patient expenditure (MBS and PBS combined) for Indigenous and non-Indigenous individuals for the first three years following diagnosis.

A stepwise generalized linear model was conducted to assess whether there was any difference in patient expenditure (MBS and PBS combined) between Indigenous and non-Indigenous patients, after accounting for differences in demographic and social characteristics. Age at diagnosis, Indigenous identification (reference group = non-Indigenous), sex (reference group = male), rurality (regional; remote; reference group = metropolitan), area-based deprivation quintile (reference group = Q1), and broad cancer site groupings (18 categories: head and neck; digestive organs; colorectal cancer; female genital organs; breast cancer; prostate cancer; male genital organs excluding prostate; urinary tract; eye, brain and other parts of the central nervous system; mesothelioma, Kaposi sarcoma and soft tissue; thyroid and other endocrine organs; other thoracic and respiratory organs; bone; tracheal, bronchus and lung; other skin; melanoma; blood and lymphatic system; and other or ill-defined cancers) were included as co-variates in the model. As with most cost data, patient expenditure was skewed. As such, we utilised generalised linear regression modelling, and selected a negative binomial distribution, with a log link function as the best representation of the data. In order to account for censoring due to death, with some patients living for the full thirty-six months and others living for shorter amounts of time, we also included the log of the number of months the patient survived as an offset to the model. A stepwise approach to this model was utilised to examine whether being Indigenous remained a significant predictor of expenditure when rurality, and then area-based deprivation were added to the model, as these are known to be strongly associated with access and out of pocket costs [11, 29].

Research suggests that a high proportion of Indigenous Australians live in rural/remote areas within Australia, as well as being more socio-economically disadvantaged than non-Indigenous Australians [3, 5]. To account for the possible collinearity of Indigenous status, rurality and socio-economic disadvantage, the analyses as explained above was first performed using a multiple linear regression analysis to examine collinearity. The output of this analysis revealed no significant collinearity between variables, with all VIF values < 5 and no tolerance values > 1.

### Cost of MBS broad type of service (BTOS)

We then limited our dataset to MBS data only to further examine the types of services covered by the Medicare scheme that each patient was accessing. The MBS item code of each occasion of service was assigned to a Medicare Broad Types of Service (BTOS), which is defined by the Australian Department of Health. There are 18 overarching BTOS categories of Medicare item codes, as outlined in Table 1 [30]. The authors mapped MBS item codes from the MBS dataset to 16 BTOS categories (the final two categories were non-MBS, and therefore, not included in the mapping). Items falling into the Obstetrics BTOS were excluded due to low numbers.

**Table 1:** A brief example of the types of item codes in each BTOS category [42]

BTOS Category	Examples of services included
Non-referred attendances – General Practitioner (GP)/Vocationally registered GP (VRGP) (101)	Attendance by a GP or VRGP
Non-referred attendances – enhanced primary care (102)	Health assessments; GP management plans, team care arrangements and multidisciplinary care plans; case conferences; GP mental health treatment plans; domiciliary and residential management reviews
Non-referred attendances – other (103)	Professional attendance at consulting rooms, or nursing home, or hospital, to which no other item applies; family group therapy, examination by a specialist in preparation for the administration of anaesthetic
Practice nurse items (110)	Services provided by a practice nurse or Aboriginal and Torres Strait Islander Health Practitioner on behalf of a medical practitioner
Other allied health (150)	Aboriginal and Torres Strait Islander services provided by an eligible Aboriginal health worker or Aboriginal and Torres Strait Islander Health Practitioner, dental services, diabetes education services, mental health services, physiotherapy services, etc
Specialist attendance (200)	Attendances by a consultant physician practicing in his or her own specialty, and was not limited to specialists practicing in haematology, medical oncology, radiation oncology, or surgical oncology
Anaesthetics (400)	Administration of anaesthetic for medical procedures
Pathology collection items (501)	Initiation of a patient episode by collection of a specimen
Pathology tests (502)	Included all pathology tests, such as simple basic pathology tests, chemical, haematology, immunology, microbiology, tissue pathology, and cytology
Diagnostic imaging (600)	Included all modalities (ie ultrasound, computed tomography, diagnostic radiography, magnetic resonance imaging, nuclear medicine imaging), and for all purposes (ie general, cardiac, vascular, injury, obstetric and gynaecological)
Operations (700)	Surgical procedures for any speciality, including colorectal, ear nose and throat, general, gynaecological, plastics and reconstructive, urological, vascular, etc
Assistance at operations (800)	11 item codes for which assistance was required during an operation
Optometry (900)	Initial consultations, subsequent consultations, appointments for contact lenses etc
Radiotherapy and therapeutic nuclear medicine (1000)	Included item codes for radiation oncology such as superficial, megavoltage, brachytherapy, and computerised planning; as well as therapeutic nuclear medicine such as administration of a radioisotope, or iodine etc.
Other MBS services (1100)	Other diagnostic and therapeutic procedures not listed elsewhere

A full list of MBS item codes mapped to the BTOS are available from the MBS website (www.mbsonline.gov.au)

Initial descriptive analyses were performed to examine the difference in both expenditure and frequency of use for each BTOS, stratified by Indigenous and non-Indigenous people with cancer. Generalised linear models using a log link function and a negative binomial distribution with an overset for survival were constructed, with the average expenditure or frequency of use for each BTOS category used as the outcome variable. All models were adjusted for age at diagnosis, Indigenous identification (reference group = non-Indigenous), sex (reference group = male), rurality (regional; remote; reference group = metropolitan), area-based deprivation quintile (reference group = Q1), and broad cancer site groupings (18 categories, as listed above). The adjusted ratios from these models, comparing the differences in average expenditure or frequency of use of each BTOS based on Indigenous identification were calculated.

Finally, a counterfactual analysis was undertaken to compare the actual frequency of occasions of service associated with each BTOS for Indigenous patients that would need to be supplied in a counterfactual scenario where Indigenous patients had the same frequency of services as their non-Indigenous counterparts. That is, we calculated how many services Indigenous patients would have had if they had the same level of services use as non-Indigenous patients, after adjusting for age at diagnosis, sex (reference group = male), rurality (regional; remote; reference group = metropolitan), area-based deprivation quintile (reference group = Q1), and broad cancer site groupings (18 categories, as listed above). We then estimated the counterfactual patient expenditure associated with each BTOS based upon the estimated counterfactual number of services that would have been accessed and the *actual* average patient co-payment per BTOS for Indigenous patients.

### Weighting to the Australian population

As described previously [26], we weighted the administrative data to provide results that are representative of the Australian population using the programmed SAS macro, GREGWT. The benchmark used for this study was the 2012 Australian cancer incidence rates by age and sex [31] .

All analysis was undertaken using SAS V9.4 (SAS Institute, Inc., Cary, NC, USA). Throughout the paper, weighted data is presented unless otherwise stated. All costs are reported in 2016–17 Australian dollars (AUD), which were adjusted with the Reserve Bank of Australia inflation calculator [32].

Human Research Ethics approval was obtained from the Townsville Hospital and Health Service Human Research Ethics Committee (HREC) (HREC/16/QTHS/11), Australian Institute of Health and Welfare (EO2017/1/343) and James Cook University HREC (H6678). Permission to waive individual consent was approved from Queensland Health under the Public Health Act 2005. No identifiable information was provided to the authors.

## Results

A total of 25,560 individuals were diagnosed with cancer in Queensland, Australia between July 2011 and June 2012. Once weighted, this represented 123,949 Australians (2% Indigenous and 98% non-Indigenous Australians, not age-standardised). Table 2 reports the descriptive demographic statistics of the people with cancer in CancerCostMOD at diagnosis. Compared to non-Indigenous people with cancer, a higher proportion of Indigenous people with cancer were female, lived in remote areas, and were in area-based deprivation quintiles 1 and 2. Approximately one-third of all participants diagnosed with cancer had died within 36 months of diagnosis.

**Table 2:** Descriptive demographics, new cancer diagnoses between 1 July 2011 and 30 June 2012

	Indigenous people with cancer	Non-Indigenous people with cancer
N	429 (1.7)	25,124 (98.3)
N (weighted)	2100	121,900
Died within 36 months of diagnosis (N, %)*	720 (34.4)	39,400 (32.3)
Female (N, %)**	1050 (49)	54,000 (44)
Mean age at diagnosis (SD)**	57.4 (15.4)	65.9 (15.2)
*Rurality* ^¥**^		
Metropolitan (N, %)	600 (27.5)	57,900 (47.8)
Regional (N, %)	800 (38.1)	53,700 (44.3)
Remote (N, %)	750 (34.4)	9600 (7.9)
*Area-based deprivation measure* ^¥**^		
1st quintile – most deprived (N, %)	600 (27)	10,750 (8.9)
2nd quintile (N, %)	150 (6.5)	5600 (4.6)
3rd quintile (N, %)	550 (24.5)	19,400 (16)
4th quintile (N, %)	600 (27)	55,500 (45.8)
5th quintile – least deprived (N, %)	350 (15)	30,000 (24.7)

^¥^Those with missing postcode data at diagnosis were excluded (*n* = 151). **p sig at <.01. *p sig at <.05

The total amount charged for services covered by the MBS in the 36 months following diagnosis was $18,899,737 for Indigenous patients and $1,876,091,278 for non-Indigenous patients, of this 3.5% was made up of co-payments for Indigenous patients and 7.9% was made up of co-payments for non-Indigenous patients. For prescriptions covered by the PBS, $16,144,895 was charged for Indigenous patients, with 4.6% being made up of co-payments, and $1,240,764,814 was charged for non-Indigenous patients, with 5.7% being made up of co-payments.

Both Indigenous and non-Indigenous people with cancer had higher expenditure on MBS and PBS co-payments in the twelve months immediately following diagnosis, than in the 13–24 and 25–36 months following diagnosis (Table 3). In the first 12 months post-diagnosis, the mean expenditure for non-Indigenous people with cancer was approximately $1074, which was more than double that of Indigenous people with cancer ($401). While expenditure was reduced in the 13–24 and 25–36 month periods for both Indigenous and non-Indigenous people with cancer, the large difference in expenditure between Indigenous and non-Indigenous people remained consistent during these periods.

**Table 3:** Average annual patient co-payments (MBS and PBS combined) by Indigenous and non-Indigenous people

Time since diagnosis	Indigenous Cancer Patients				Non-Indigenous Cancer Patients			
	N	Mean (SD)	Median (IQR)	Range	N	Mean (SD)	Median (IQR)	Range
0 to 12 months	1660	401 (817)	177 (425)	0–8568	97,200	1074 (1986)	450 (831)	0–81,814
13 to 24 months	1480	200 (455)	67 (221)	0–6180	88,200	484 (876)	289 (404)	0–25,731
25 to 36 months	1380	181 (421)	61 (226)	0–6046	83,100	441 (825)	269 (382)	0–32,691

Note: data is limited to those who survived each respective 12-month period

The abbreviated output of the parameter estimates produced from the stepwise generalised linear model of patient expenditure for MBS and PBS combined in the first thirty-six months following diagnosis are shown in Table 4 (broad cancer type is not shown, but is included in the model). Regardless of the other demographic and social characteristics being adjusted for, identifying as Indigenous remained a significant predictor of patient expenditure at each stage of the model. The co-efficient values suggest that Indigenous people with cancer spent significantly less on direct expenditure than their non-Indigenous counterparts. In the final model, those in area-based deprivation quintiles four and five paid significantly more than those in quintile one (the most disadvantaged quintile), while age at diagnosis was also a significant predictor.

**Table 4:** Parameter estimates of independent variables in stepwise generalised linear regression model of annual patient co-payment^1,2^

Variable	Model 1: Sex, age and Indigenous identification only			Model 2: Sex, age, Indigenous identification + Rurality			Model 3: Sex, age, Indigenous identification + Rurality + Disadvantage		
	*Co-efficient*	*SE*	*p-value*	*Co-efficient*	*SE*	*p-value*	*Co-efficient*	*SE*	*p-value*
Intercept	3.98	0.05	<.0001	4.05	0.05	<.0001	3.81	0.06	<.0001
Female	−0.03	0.02	0.18	−0.03	0.02	0.13	−0.04	0.02	0.52
Age at diagnosis	0.007	0.001	<.0001	0.007	0.001	<.0001	0.01	0.001	<.0001
Indigenous identification	−1.03	0.07	<.0001	−1.01	0.07	<.0001	−0.98	0.07	<.0001
Regional Area				−0.15	0.02	<.0001	−0.04	0.02	0.11
Remote Area				−0.16	0.03	<.0001	−0.01	0.04	0.83
Area-based deprivation Quintile 2							−0.04	0.05	0.39
Area-based deprivation Quintile 3							0.01	0.04	0.71
Area-based deprivation Quintile 4							0.19	0.04	<.0001
Area-based deprivation Quintile 5							0.30	0.05	<.0001

^1^MBS and PBS patient co-payments combined

^2^Abbreviated output, all models adjusted for cancer type

The average frequency of use for each BTOS service for Indigenous and non-Indigenous individuals in this dataset is shown in Table 5 along with the adjusted ratio in number of services utilised. There was no significant difference in the number of GP attendances based on whether people with cancer identified as Indigenous, however Indigenous people with cancer had 38% more enhanced primary care visits, 54% more services with a practice nurse, and 45% more other non-referred primary care services than non-Indigenous people with cancer. In contrast, Indigenous people with cancer had 49% fewer specialist attendances than non-Indigenous people with cancer. Indigenous people with cancer also had 22% fewer pathology collection services, 32% fewer pathology tests, and 25% fewer diagnostic imaging services.

**Table 5:** Average number and adjusted ratio of Medicare services by Indigenous status

BTOS name	BTOS code	Mean (SD)		Ratio in number of services between Indigenous and non-Indigenous people with cancer^a^
		Indigenous	Non-indigenous	
Non-referred attendances – GP/VRGP	101	29 (25.8)	33.5 (26.8)	1.04
Non-referred attendances – enhanced primary care	102	6.2 (6.4)	5.3 (5.1)	1.38***
Non-referred attendances – other	103	5.4 (7.9)	4.4 (7.7)	1.45***
Non-referred attendances – practice nurse items	110	4.5 (6.8)	3.4 (4.4)	1.54***
Other allied health	150	10 (12.5)	10.2 (10.6)	1.07
Specialist attendances	200	14 (19.5)	28.6 (41.8)	0.51***
Anaesthetics	400	2.9 (2.3)	4.5 (4.5)	0.62***
Pathology collection items	501	32.2 (37.1)	46.2 (50.2)	0.78***
Pathology tests	502	38.7 (45.5)	61.3 (85.3)	0.68***
Diagnostic imaging	600	10.7 (9.9)	14.3 (11.9)	0.75***
Operations	700	6.1 (8.9)	9.2 (10.7)	0.71***
Assistance at operations	800	1.5 (0.9)	1.6 (1.1)	0.90
Optometry	900	2.4 (2.2	2.7 (2.5)	0.97
Other MBS Services	1000	37.2 (20.8)	39 (25.3)	0.93
Radiotherapy and therapeutic nuclear medicine	1100	34.9 (36.8)	44 (50.4)	1.02
**All BTOS combined**		**177.3 (149.4)**	**248.5 (234)**	0.80***

^#^Adjusted for age at diagnosis, Indigenous identification, sex, rurality, area-based deprivation quintile, and broad cancer site groupings

*significant at 0.05 level

**significant at 0.01 level

***significant at 0.001 level

The expenditure associated with each BTOS in the three years following diagnosis for Indigenous and non-Indigenous people with cancer is shown in Table 6, along with the adjusted cost ratio of expenditure for each BTOS. This suggests that Indigenous patients spend significantly less on the majority of health services than non-Indigenous patients. Despite Table 5 showing Indigenous people with cancer access more or similar numbers of non-referred services, Indigenous people with cancer spent 58% less on GP services than non-Indigenous people with cancer, 99.1% less on enhanced primary care services, and 50% less on services in the ‘other’ category.

**Table 6:** Average expenditure and adjusted cost ratio of Medicare service types by Indigenous status

BTOS name	BTOS code	Mean (SD) co-payment		Adjusted Cost Ratio^#^
		Indigenous	Non-indigenous	
Non-referred attendances – GP/VRGP	101	73.4 (197)	162.6 (286)	0.42***
Non-referred attendances – enhanced primary care	102	0.05 (0.9)	2 (19.2)	0.009***
Non-referred attendances – other	103	22.8 (126.9)	46.9 (120.8)	0.50*
Non-referred attendances – practice nurse items	110	0.09 (1.1)	0.2 (2)	0.11
Other allied health	150	19.9 (93.9)	58.9 (183.8)	0.33**
Specialist attendances	200	260.3 (670.6)	978.3 (1650.2)	0.25***
Anaesthetics	400	580.9 (658.6)	1005.5 (1097.5)	0.54***
Pathology collection items	501	10.8 (48.1)	42.1 (128.2)	0.23***
Pathology tests	502	109.5 (440.5)	481.1 (1215.2)	0.21***
Diagnostic imaging	600	112.9 (329)	400.4 (768.6)	0.26***
Operations	700	671.3 (1463.7)	1797.9 (2510.7)	0.39***
Assistance at operations	800	366.8 (303.3)	392.1 (345.1)	0.83
Optometry	900	0.6 (4.9)	0.9 (5.7)	2.24
Other MBS Services	1000	159.4 (702.7)	682.7 (1487.2)	0.14***
Radiotherapy and therapeutic nuclear medicine	1100	85.8 (495.3)	375.8 (1233.9)	0.26***
**All BTOS combined**		**1191 (3099)**	**4639 (6891)**	0.25***

^#^Adjusted for age at diagnosis, sex, rurality, area-based deprivation quintile, and broad cancer site groupings

*significant at 0.05 level

**significant at 0.01 level

***significant at 0.001 level

Table 6 shows that Indigenous people with cancer also had lower expenditure in total on these services. For example, non-Indigenous people with cancer spent an average of $978 on specialist patient co-payments in the three years post diagnosis, whereas Indigenous people with cancer spent an average of $260 on co-payments for this type of service. After adjusting for age, sex, type of cancer, rurality and area-based deprivation, Indigenous people with cancer spent 75% less on specialist services, 79% less for pathology tests, 74% less for diagnostic imaging and 61% less for operations. Overall Indigenous people with cancer spent 75% less on all services in the three years following their diagnosis than non-Indigenous people with cancer.

Table 7 shows the actual number of BTOS services accessed by Indigenous Australians, and the counterfactual number of BTOS services that would be accessed if Indigenous Australians had utilised services at the same rate as their non-Indigenous counterparts. For all BTOS services combined, Indigenous Australians accessed an average of 236 services, but if Indigenous Australians had the same rate of service use as their non-Indigenous counterparts, the estimated number of services would increase to an average of 309 services per person, resulting in an estimated expenditure on co-payments of $3242.50. The actual average expenditure (shown in Table 6) on all BTOS services combined was $1191 for Indigenous people and $4639 for non-Indigenous people.

**Table 7:** Estimated number of services if Indigenous people had equal access to non-Indigenous people

BTOS	BTOS code	Actual average number of services accessed	Actual average cost of services accessed	Counterfactual average number of services if access was equal	Estimated average patient co-payment if access was equal
Non-referred attendances – GP/VRGP	101	29	$73	33	$83.5
Non-referred attendances – enhanced primary care	102	6	$0.05	4	$0.03
Non-referred attendances – other	103	5	$23	4	$17
Non-referred attendances – practice nurse items	110	4	$0.09	3	$0.06
Other allied health	150	10	$20	9	$17.9
Specialist attendances	200	14	$260	30	$561.8
Anaesthetics	400	3	$581	4	$815.3
Pathology collection items	501	32	$11	47	$15.7
Pathology tests	502	39	$110	67	$189.5
Diagnostic imaging	600	11	$113	16	$168.7
Operations	700	6	$671	8	$877.5
Assistance at operations	800	2	$367	1	$241.3
Optometry	900	2	$0.6	2	$0.5
Other MBS Services	1000	37	$159	39	$167.2
Radiotherapy and therapeutic nuclear medicine	1100	35	$86	42	$103.2
**ALL BTOS combined**		**236**	**$1191**	**309**	**$3242.5**

## Discussion

Ensuring equitable access lies at the core of universal health care [33]. A significant part of this is ensuring that out of pocket costs associated with accessing health care do not cause financial distress, and as such do not affect individual demand for, and access to health care services. The initial results of this study indicated that Indigenous people with cancer in Australia pay significantly less on co-payments for services covered under Medicare than non-Indigenous people with cancer in the first three years after diagnosis. However, this lower amount of expenditure was largely driven by differences in patterns of service access between the two groups, as well as lower co-payments for services for Indigenous people with cancer.

Our results have shown that overall, Indigenous people with cancer are accessing fewer services covered by Medicare. This includes fewer pathology tests and diagnostic imaging services, as well as specialists, and radiotherapy and therapeutic nuclear medicine services. It may be that some Indigenous people with cancer are accessing some specialist and radiotherapy services in outpatient clinics at public hospitals. While many of these services are billed through Medicare, in which case they would be included in the results of this study, it is also possible for such services to be funded by state and territory health budgets and so would not be captured in our data [34]. Indigenous people with cancer do access more primary care services, such as services provided by General Practitioners, than non-Indigenous people with cancer. It is important to note that Indigenous people with cancer are likely to have more co-morbidities [4], which may explain this, at least in part.

In order to account for these differences in access patterns, we modelled a counterfactual scenario whereby Indigenous people with cancer had the same rate of access to services covered under Medicare as non-Indigenous people with cancer with otherwise the same demographic and clinical characteristics. Results from this modelling indicated that Indigenous people with cancer would incur much higher (compared to their actual expenditure) out of pocket expenditure for co-payments, an increase from $1191 to $3243 over the three years following diagnosis. The latter figure is still less than the $4639 paid by non-Indigenous people with cancer, reflecting the lower co-payment amounts for individual occasions of services paid by Indigenous people with cancer.

These findings indicate that when Indigenous people do access services, that Australia’s universal health care policies, such as the Extended Medicare Safety Net and Closing the Gap (CTG) Closing the Gap (CTG) PBS Co-payment Programme, are supporting equity. Those with greater need – Indigenous people with cancer have known poorer survival outcomes – are receiving a greater amount of financial re-imbursement. However, the findings suggest substantial inequity in terms of access to services – with Indigenous people with cancer accessing far fewer services. These findings have important policy implications, with universal healthcare being listed as one of the Sustainable Development Goals (SDG) [35], which have been adopted by 150 world governments including Australia. Australia’s ranking on the SDG Index has declined between 2016 and 2018 from 26th to 37th [36], highlighting the need for improvement in all domains, including health. Yet developed countries, with long-established universal health care systems, such as Australia, are having the sustainability of their system questioned domestically, particularly in light of tighter fiscal policy environments following the Global Financial Crisis [37]. Furthermore, within Australia, significant attention has been given to the actual performance of Australia’s universal healthcare system [19, 38, 39]. Differences in access to services based upon socioeconomic status or location have been previously documented [40, 41]. Our findings have shown that this pattern is also true for Indigenous people with cancer.

This analysis does have a number of limitations that need to be considered. Primarily the costs were limited to co-payments for services listed under the MBS and prescriptions for pharmaceuticals listed on the PBS. Payment for services not covered under these schemes have not been included. Furthermore, indirect cost associated with travel and accommodation, childcare, lost wages from employment, and intangible costs such as time away from family are also not included and as such we underestimate the full costs to people with cancer. We were also unable to identify any private health insurance rebates that may have been paid for MBS services delivered in private hospitals. As there is overlap between Indigenous status, rurality and socioeconomic disadvantage within Australia, the unique contribution that each factor played in predicting cancer costs was not fully explored using the analysis in this paper. Finally, the analysis was unable to adjust for co-morbidities, which may have been higher in Indigenous people with cancer; or for educational level.

## Conclusion

While this study did find that Indigenous people with cancer do pay less out of pocket for co-payments associated with accessing care, it also highlighted deep inequalities in terms of access to healthcare services provided under Medicare. These inequalities in access with regards to equity in out of pocket payments between Indigenous and non-Indigenous people with cancer, must also be considered within in the context of the other deep structural inequities that inhibit Indigenous people’s access to care through impacting clinician or patient-side decision-making. This study has highlighted the challenges of assessing out of pocket expenditure. While it appears that Indigenous people with cancer do pay less when they do access services, overall they access far fewer services than non-Indigenous people with cancer. It appears that this lower access may be having a negative impact given the well-documented disparities in cancer outcomes for Indigenous people with cancer.

## Abbreviations

ABS: Australian Bureau of Statistics
ASGS: Australian Statistical Geography Standard
BTOS: Broad Types of Service
CTG: Closing the Gap
EDIS: Emergency Department Information System
GP: General Practitioner
HREC: Human Research Ethics Committee
IRSD: Index of Relative Socio-Economic Disadvantage
MBS: Medicare Benefits Schedule
PBS: Pharmaceutical Benefits Scheme
QCR: Queensland Cancer Registry
QHAPDC: Queensland Health Admitted Patient Data Collection

## Footnotes

1 However, hospital prescriptions are excluded from the CTG PBS Co-Payment Programme.
